# Protein phosphatase 4 coordinates glial membrane recruitment and phagocytic clearance of degenerating axons in *Drosophila*

**DOI:** 10.1038/cddis.2017.40

**Published:** 2017-02-23

**Authors:** Lilly M Winfree, Sean D Speese, Mary A Logan

**Affiliations:** 1 Jungers Center for Neurosciences Research, Department of Neurology, Oregon Health and Science University, 3181 SW Sam Jackson Park Road, Portland, OR 97239, USA

## Abstract

Neuronal damage induced by injury, stroke, or neurodegenerative disease elicits swift immune responses from glial cells, including altered gene expression, directed migration to injury sites, and glial clearance of damaged neurons through phagocytic engulfment. Collectively, these responses hinder further cellular damage, but the mechanisms that underlie these important protective glial reactions are still unclear. Here, we show that the evolutionarily conserved trimeric protein phosphatase 4 (PP4) serine/threonine phosphatase complex is a novel set of factors required for proper glial responses to nerve injury in the adult *Drosophila* brain. Glial-specific knockdown of PP4 results in reduced recruitment of glia to severed axons and delayed glial clearance of degenerating axonal debris. We show that PP4 functions downstream of the the glial engulfment receptor Draper to drive glial morphogenesis through the guanine nucleotide exchange factor SOS and the Rho GTPase Rac1, revealing that PP4 molecularly couples Draper to Rac1-mediated cytoskeletal remodeling to ensure glial infiltration of injury sites and timely removal of damaged neurons from the CNS.

Glial cells continuously survey the brain and respond swiftly to any form of stress or damage.^1^ Acute insults, as well as chronic neurodegenerative conditions, trigger robust immune responses from glia.^2, 3^ Reactive glia undergo striking morphological changes and infiltrate injury sites to rapidly phagocytose cellular debris.^4, 5^ Glial changes in cell shape, size, and migration in response to neurodegeneration are highly conserved hallmark reactions to trauma in species ranging from *Drosophila* to humans. Following injury, glial cells either migrate to injury sites or, in instances where the cell soma remains in a fixed location, reactive glia send dynamic membrane projections into regions that house damaged neurons.^5, 6, 7^ Importantly, inhibiting these glial morphogenic responses delays phagocytic clearance of neurotoxic cellular debris, which can attenuate postinjury neuronal plasticity, trigger secondary inflammatory reactions, and exacerbate damage.^8, 9^ Despite the fact that glial cells undergo significant changes in size and shape to access trauma sites and clear damaged cells, the molecular mechanisms responsible for glial migration and directed extension of processes are not entirely understood.

Acute axotomy of the olfactory nerve in adult *Drosophila melanogaster* is a well-established injury paradigm to investigate the molecular mechanisms that govern glial morphogenesis and phagocytic function in response to axon degeneration.^4, 5, 10, 11, 12, 13, 14, 15, 16^ *Drosophila* glia are morphologically and functionally similar to mammalian glia, and fly glial responses to axon injury mirror those that occur in vertebrate models.^4, 17, 18, 19^ After severing adult maxillary palp or antennal olfactory nerves, local ensheathing glia extend membrane projections to infiltrate antennal lobe neuropil and phagocytose degenerating axonal debris; glial invasion of the antennal lobes requires the highly conserved glial immune receptor Draper/MEGF10.^5^ Activated Draper signals via Src family kinases, which leads to activation of Rac1 and cytoskeletal remodeling.^13, 14, 15, 16, 20^ Recent work has identified two guanine nucleotide exchange factors (GEFs), Crk/Mbc/Ced-12 and DRK/DOS/SOS, that activate Rac1 in this context, directly associating with Rac1 to catalyze the exchange of GDP for GTP,^13, 16^ but we still have a poor understanding of the molecular effectors that couple the transmembrane receptor Draper to Rac1-mediated cytoskeletal changes.

The evolutionarily conserved serine/threonine protein phosphatase 4 (PP4) complex is involved in diverse cellular functions, including cell proliferation and apoptosis, and is required for embryonic development across species.^21, 22, 23, 24, 25, 26, 27, 28^ The PP4 complex consists of three subunits: one catalytic subunit (PP4c), which is required for dephosphorylation of target proteins, and two regulatory subunits (PP4r2 and Falafel/PP4r3), which control subcellular localization of the complex and specificity of phosphatase target association.^21, 29^ Interestingly, PP4 is also linked to cell motility and tumor invasiveness in several organisms and cell types. For example, in the slime mold *Dictyostelium discoideum*, PP4 phosphatase activity is necessary for chemotaxis, and in human colorectal carcinoma cells, active PP4 promotes cell migration.^25, 30^ PP4 is also a positive regulator of Rac1-dependent cell movement in cultured HEK293 cells.^30, 31^ The role of the PP4 complex in glial responses to neural injury, however, has never been explored. Here, we demonstrate a novel role for PP4 in glia as they respond to severed axons. We propose that PP4 is a downstream effector of the glial receptor Draper and that it signals through the SOS GEF complex and the GTPase Rac1 to promote proper glial membrane infiltration of injury sites and clearance of degenerating neuronal material.

## Results

### PP4 is required for proper glial clearance of severed axons

**Figure 1:**
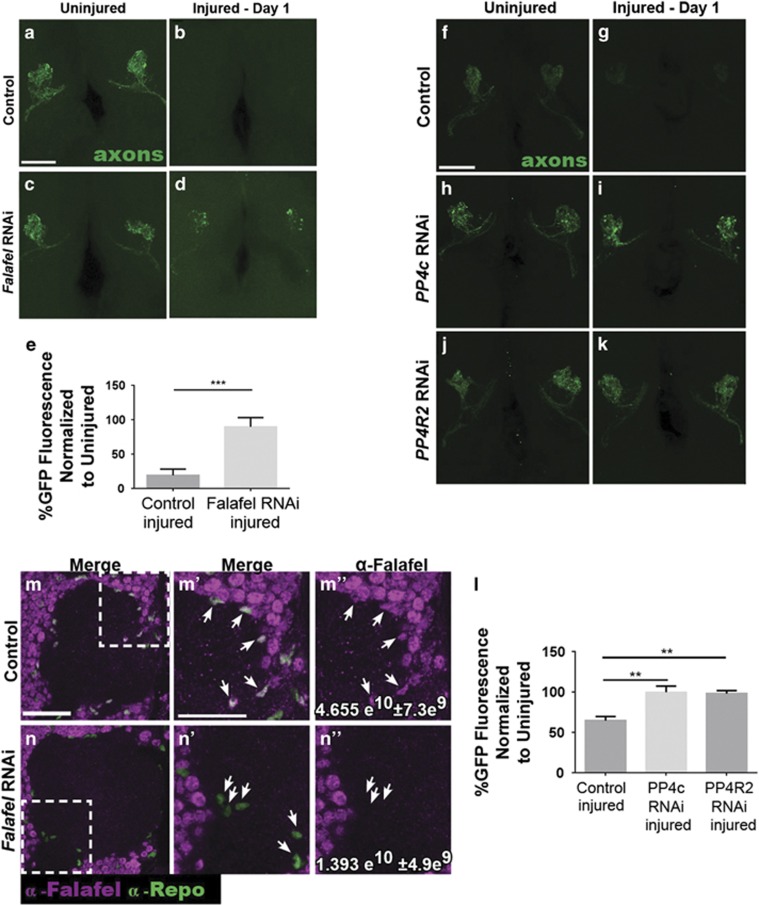
The PP4 complex subunits Falafel, PP4c, and PP4r2 are required for glial clearance of degenerating axons. (**a–d**) Representative maximum intensity projection confocal images (z-stack, 15 *μ*m) show green fluorescent protein (GFP)-labeled OR85e axonal projections (green) in antennal lobes of uninjured (**a** and **c**) and injured (**b** and **d**) adult flies. (**e**) Quantification of OR85e GFP fluorescence from experiment in panels (**a**–**d**) normalized to uninjured; mean±S.E.M. plotted; one-way analysis of variance (ANOVA). ***P<0.001. (**f–k**) Representative maximum intensity projection images (z-stack, 15 *μ*m) shown in antennal lobes of uninjured (**f**, **h**, and **j**) and injured (**g**, **i** and **k**) adult flies. (**l**) Quantification of OR85e axonal debris (GFP fluorescence) from experiment in panels (**f**–**k**) normalized to uninjured conditions; mean±S.E.M. plotted; one-way ANOVA. **P<0.01. (**m–n**) Representative antennal lobe regions (*z*=3 *μ*m) show cells stained with anti-Falafel (magenta) and anti-Repo (glia nuclear marker, green) in control (**m**) and Falafel RNAi (**n**). Falafel fluorescence intensity in Repo+ glia nuclei was quantified from the entire central brain. See values (white font) in panels (**m**″ and **n**″). Unpaired *t*-test. *P*<0.0001. Scale bars=20 *μ*m. Genotypes: Control=*OR85e-mCD8::GFP,tub-Gal80*^*ts*^*/+ repo-Gal4/+.* Falafel RNAi=*OR85e-mCD8::GFP,tub-Gal80*^*ts*^*/+ repo-Gal4/UAS-falafel*^*RNAi*^. *RNAi=OR85e-mCD8::GFP,tub-Gal80*^*ts*^*/UAS-PP4c*^*RNA*^*i; repo-Gal4/+. PP4r2 RNAi=OR85e-mCD8::GFP,tub-Gal80*^*ts*^*/UAS-PP4r2*^*RNAi*^*; repo-Gal4/+*

To inhibit PP4 function in adult *Drosophila* glia, we expressed *UAS-falafel*^*RNAi*^ with the pan-glial driver *repo-Gal4* and assayed clearance of degenerating olfactory receptor neuron (ORN) axons. Importantly, because PP4 phosphatase activity is critical for proper development, including asymmetric localization of cell fate determinants in larval neuroblasts,^32^ these flies also carried the *tubulin-Gal80*^*ts*^ transgene, which allowed us to temporally regulate GAL4 activity and express *falafel*^*RNAi*^ specifically in adult glia.^33^ To monitor clearance of axonal debris, a subset of maxillary palp ORNs were labeled with membrane-tethered GFP (*OR85e-mCD8::GFP*). We severed the maxillary nerves that project into the antennal lobes and then quantified axonal GFP+ fluorescence in each OR85e glomerulus one day after axotomy using previously published methods.^4, 11, 12^ In controls, most GFP+ axonal debris was cleared within 1 day (Figures 1a and b). In *falafel*^*RNAi*^ flies, significantly more GFP+ axonal material was present 1 day postaxotomy (Figures 1c–e, *P*<0.001), suggesting that Falafel is necessary for proper glial engulfment of severed axons. To confirm efficacy of *falafel*^*RNAi*^, we performed immunostaining against Falafel and the glial-specific transcription factor Repo on adult brains. In control animals, Falafel appeared to be localized, or enriched, in glial nuclei (Figure 1m), and we detected a 70% reduction of glial nuclear Falafel fluorescence in *falafel*^*RNAi*^ flies (Figures 1m and n).

In addition to Falafel, the PP4 complex contains a second regulatory subunit, PP4r2, and a catalytic phosphatase subunit, PP4c. To determine if a complete PP4 complex is necessary for proper glial engulfment of debris, we again used the *Gal4/Gal80*^*ts*^ system to knockdown PP4c (*UAS-PP4c*^*RNAi*^) and PP4r2 (*UAS-PP4r2*^*RNAi*^) independently in adult glia. One day after severing maxillary nerves, we observed significantly more OR85e GFP+ axonal debris lingering in the antennal lobes in *PP4c*- and *PP4r2*-depleted flies (Figures 1f–l, *P*<0.01). To confirm efficacy of our *Gal4/Gal80*^*ts*^ experiments, we repeated these clearance assays while maintaining flies at the permissive temperature of 22 °C, and observed normal clearance (Supplementary Figures 1a–i). Finally, we performed a short time course to assess clearance in *PP4c*-depleted flies and found that significantly more axonal material persisted in the brain for at least a week after axotomy (Supplementary Figure 1j). Taken together, these results indicate that the PP4 serine/threonine phosphatase complex is essential for efficient glial engulfment of axonal debris in the adult brain.

### PP4 is essential for proper recruitment of Draper and glial membranes to severed axons

**Figure 2:**
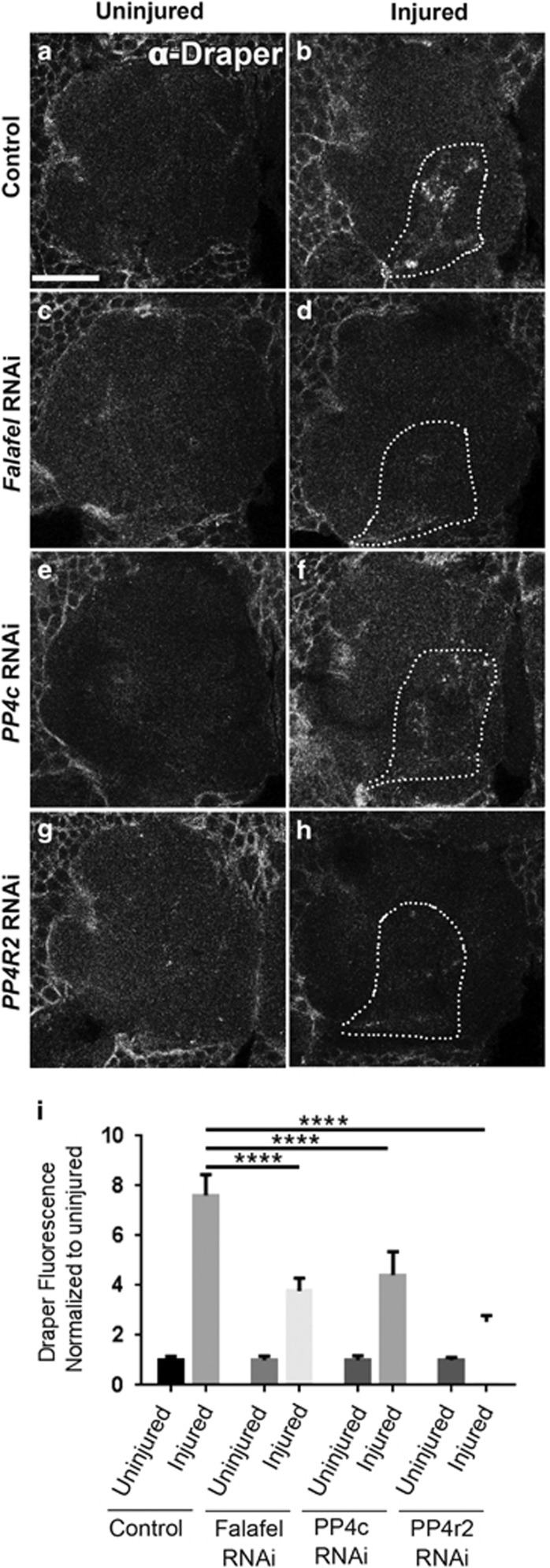
PP4 is required for proper recruitment of Draper to degenerating axons. (**a–h**) Representative single z-slice (1 *μ*m) show anti-Draper fluorescence in one antennal lobe of uninjured (**a**, **c**, **e**, and **g**) and injured (**b**, **d**, **f** and **h**) adult flies. White dotted outlines show OR85e glomeruli (visualized with OR85e-mCD::GFP, not shown) and representative areas of Draper fluorescence quantification. (**i**) Draper fluorescence quantified in z-stack of 15 *μ*m, normalized to uninjured conditions. Uninjured Draper set at a value of 1; mean±S.E.M. plotted; one-way analysis of variance (ANOVA). ****P<0.0001. Scale bars=20 *μ*m. Genotypes: Control=*OR85e-mCD8::GFP,tub-Gal80*^*ts*^*/+ repo-Gal4/+. Falafel RNAi=OR85e-mCD8::GFP,tub-Gal80*^*ts*^*/+ repo-Gal4/UAS-falafel*^*RNAi*^*. PP4c RNAi=OR85e-mCD8::GFP,tub-Gal80*^*ts*^*/UAS-PP4c*^*RNAi*^*; repo-Gal4/+. PP4r2 RNAi=OR85e-mCD8::GFP,tub-Gal80*^*ts*^*/UAS-PP4r2*^*RNAi*^*; repo-Gal4/+.* GFP, green fluorescent protein

Following ORN axotomy, local ensheathing glial cells robustly upregulate the Draper receptor and extend their membranes into the antennal neuropil regions to phagocytose axonal debris,^5^ and, in fact, Draper is essential for these cells to invade the neuropil and access degenerating nerves.^5, 16^ To determine if recruitment/accumulation of Draper and glial membranes was altered in PP4-depleted flies, we first expressed RNAi against Falafel (*UAS-falafel*^*RNAi*^), PP4c (*UAS-PP4c*^*RNAi*^), or PP4r2 (*UAS-PP4r2*^*RNAi*^) in adult glia using the *Gal4/Gal80*^*ts*^ system, severed maxillary nerves, and then immunostained brains for Draper. In controls, one day after maxillary nerve axotomy, we observed a significant increase in Draper on maxillary ORN-innervated glomeruli (Figures 2b and i), but this response was significantly attenuated in *falafel*^*RNAi*^, *PP4c*^*RNAi*^, and *PP4r2*^*RNAi*^ animals (Figures 2d, f, h, and i *P*<0.0001), suggesting that the PP4 complex is essential for proper recruitment of Draper to severed nerves.

**Figure 3:**
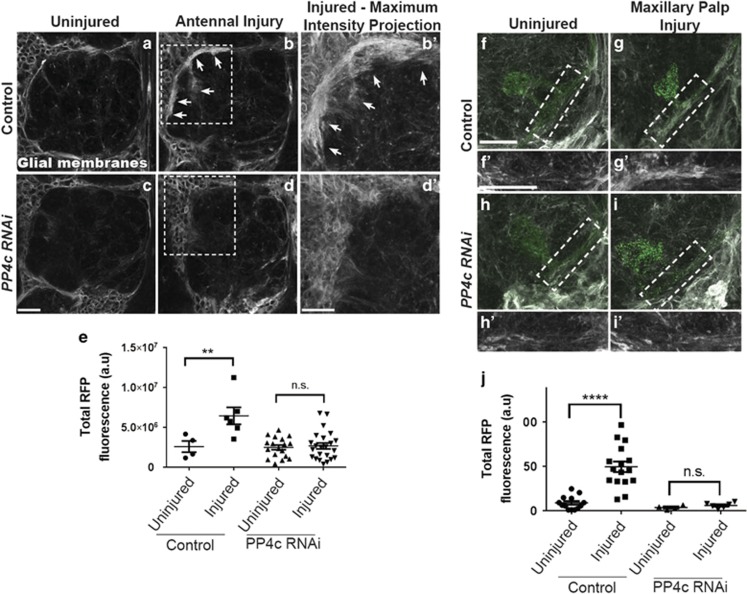
PP4 is necessary for glial membrane expansion and recruitment to severed axons after nerve injury. (**a–d**) Representative antennal lobe z-stacks (3 *μ*m) show glial membrane RFP in gray scale in uninjured (**a** and **c**) and injured (**b** and **d**) adult flies. (**b′** and **d′**) Zoomed in view of white boxed regions in (**b**) and (**d**). (**e**) Quantification of RFP+ glial membrane fluorescence, normalized to uninjured condition. Uninjured RFP fluorescence set to 1; N: control: 4, control injured: 6, PP4c: 18, PP4c injured: 24; individual data points with mean±S.E.M. plotted; one-way analysis of variance (ANOVA). **P<0.01. (**f–i**) Representative antennal lobe z-stacks (15 *μ*m) show RFP-labeled glial membranes (gray scale) and OR85e (green) in uninjured (**f** and **h**) and injured (**g** and **i**) adult flies. (**f′**, **g′**, **h′**, and **i′**) Zoomed in view of blue boxed regions in (**f–i**) show glial membrane accumulation on severed maxillary nerves in control animals (**g′**). (**j**) RFP membrane fluorescence quantified, normalized to uninjured condition. Uninjured RFP fluorescence set to 1; individual data points with mean±S.E.M. plotted; one-way ANOVA. ****P<0.001. Scale bars=20 *μ*m. Genotypes: Control=*OR85e-mCD8::GFP,tub-Gal80*^*ts*^*/+, repo-Gal4/repo-LexA, LexAop-mCD2::RFP. PP4c RNAi=OR85e-mCD8::GFP,tub-Gal80*^*ts*^*/PP4c*^*RNAi*^*; repo-Gal4/repo-LexA, LexAop-mCD2::RFP*. GFP, green fluorescent protein; NS, nonsignificant

Next, because Draper accumulation on injured nerves is tightly coupled to the process of glial infiltration of neuropil, we assessed glial membrane responses in control and PP4c knockdown flies. To visualize glial membranes, we used a fly line expressing membrane-tethered red fluorescent protein (RFP) (*UAS-mCD4::RFP*) under the control of *repo-Gal4*, and again used the *tubulin-Gal80*^*ts*^ system to express *PP4c*^*RNAi*^ specifically in adult glia. One day after antennal nerve axotomy, we observed a striking increase in ensheathing glial membrane RFP+ fluorescence around the antennal lobes (RFP in gray scale, Figure 3b and b′, *P*<0.01), which represents expansion of responding glial membranes.^5^ Importantly, in flies expressing glial *PP4c*^*RNAi*^, there was no detectable increase of glial membrane RFP after injury (Figures 3d and d′). Similarly, while maxillary palp ablation resulted in accumulation of glial membranes on the maxillary nerves of control animals (Figures 3f–g′, *P*<0.0001), we did not detect an increase in glial membrane expansion along injured maxillary nerves in *PP4c*^*RNAi*^ flies (Figures 3h–j). Finally, in uninjured flies, we did not observe any obvious changes in gross glial morphology following PP4 knockdown, nor did we detect a decrease in glial cell numbers (Supplementary Figure 2), further indicating that glial cell development is not overtly affected in these animals. Taken together, these results indicate that the PP4 complex is required in adult glia to activate a program that drives dynamic glial membrane responses to axotomy.

### PP4 functions downstream of Draper

Simply reducing basal levels of glial Draper is sufficient to delay Draper recruitment to severed nerves and clearance of axon debris in the adult brain.^5, 34^ To determine if basal Draper levels were altered in PP4 knockdown flies, we quantified Draper in the areas immediately adjacent to the antennal lobes in uninjured control flies and animals expressing *Falafel*^*RNAi*^, *PP4c*^*RNAi*^, or *PP4r2*^*RNAi*^ in adult glia. Draper levels were unchanged in *Falafel*^*RNAi*^ and *PP4r2*^*RNAi*^ flies (Supplementary Figure 3a). Unexpectedly, basal levels of Draper were increased in *PP4c^RNAi^* flies based on immunostaining (Supplementary Figure 3a) and western blot analysis (Supplementary Figures 3b and c).

**Figure 4:**
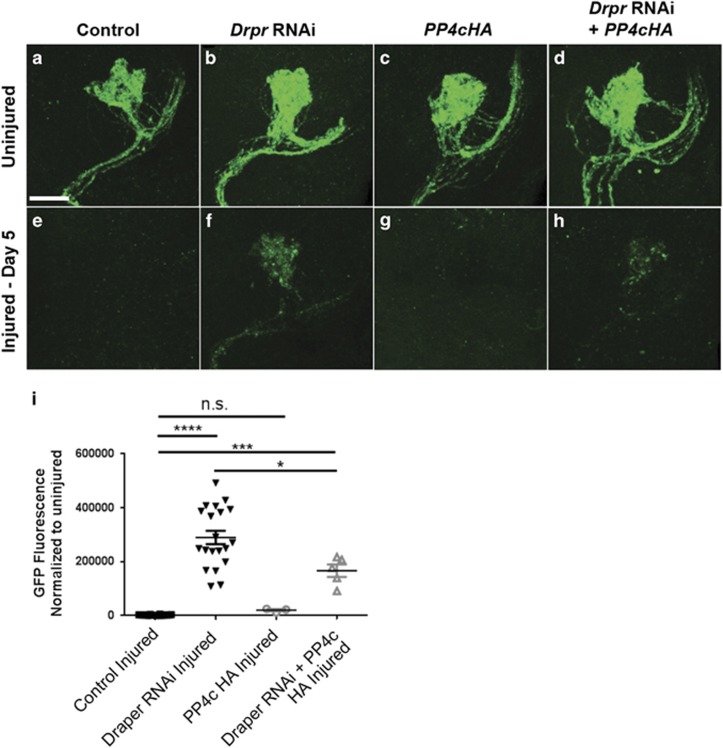
PP4c overexpression partially reverses axon clearance defects in *draper^RNAi^* animals. (**a–h**) Representative maximum intensity projection confocal images (z-stack, 15 *μ*m) show green fluorescent protein (GFP)-labeled OR85e axonal projections (green) in antennal lobes of uninjured (**a**–**d**) and injured (**e**–**h**) control (**a** and **e**), Draper RNAi alone (**b** and **f**), PP4cHA alone (**c**and **g**), Draper RNAi+PP4cHA (**d** and **h**). (**i**) Quantification of OR85e axonal debris (GFP) normalized to uninjured conditions. Uninjured GFP fluorescence values set at 1. Individual data points with mean±S.E.M. plotted; one-way analysis of variance (ANOVA). *P<0.05, ***P<0.001, and ****P<0.0001. Scale bar=20 *μ*m. Genotypes: control=*OR85e-mCD8::GFP,tub-Gal80*^*ts*^*/+ repo-Gal4/+. Drpr RNAi=OR85e-mCD8::GFP,tub-Gal80*^*ts*^*/UAS-draper^RNAi^; repo-Gal4/UAS-LacZ. PP4cHA=OR85e-mCD8::GFP,tub-Gal80*^*ts*^*/UAS-LacZ; repo-Gal4/UAS-PP4cHA. Drpr RNAi+PP4cHA=OR85e-mCD8::GFP,tub-Gal80*^*ts*^*/UAS-draper^RNAi^; repo-Gal4/UAS-PP4cHA*. NS, nonsignificant

Because PP4 inhibition did not lower Draper levels in the adult brain, we reasoned that the PP4 complex may function downstream of Draper to promote glial infiltration of antennal lobes and clearance of severed olfactory axons. To further explore this, we overexpressed PP4c (*UAS*-*PP4cHA*) and *draper^RNAi^* in adult glia and assayed OR85e axonal clearance. Glial depletion of draper significantly inhibits clearance of severed axons as compared with controls^5^ (Figures 4a, b, e, f, and i, *P*<0.0001). Glial expression of PP4c partially, but significantly, reversed this clearance defect in *draper^RNAi^* flies (Figures 4d, h, and i, *P*<0.05), suggesting that boosting PP4c can partially bypass the requirement for Draper in this injury paradigm.

### PP4 is dispensable for injury-induced activation of STAT92E in ensheathing glia

**Figure 5:**
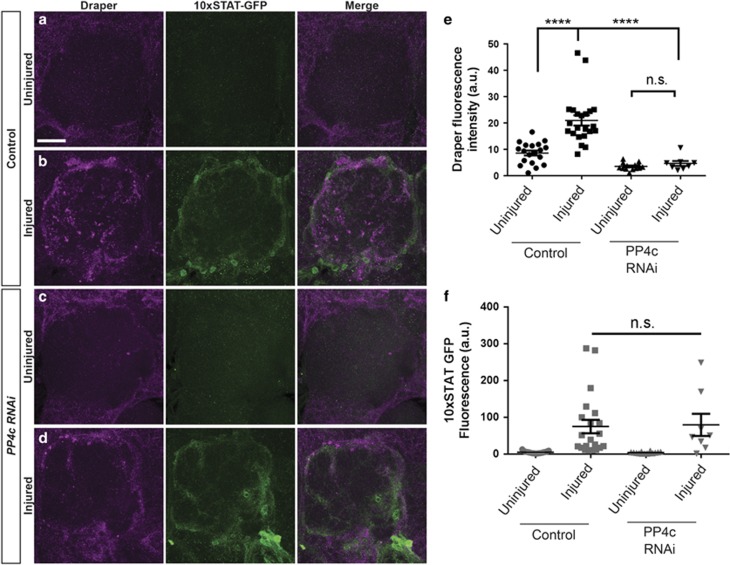
Injury-induced activation of STAT92E in ensheathing glia does not require PP4c. (**a–d**) Expression of the STAT92E transcriptional reporter *10XSTAT92E-dGFP*. Representative z-stacks (10 *μ*m) show anti-green fluorescent protein (GFP) (green) and anti-Draper fluorescence (magenta) in one antennal lobe of uninjured (**a** and **c**) and injured (**b** and **d**) adult flies. (**e**) Quantification of Draper, normalized to uninjured conditions. Uninjured Draper set at a value of 1. (**f**) Quantification of dGFP levels, normalized to uninjured conditions. Uninjured dGFP set at a value of 1. Individual data points with mean±S.E.M. plotted; one-way analysis of variance (ANOVA), ****P<0.0001. Scale bar=20 *μ*m. Genotypes: Control=*10XSTAT92E-dGFP,tub-Gal80*^*ts*^*/+ repo-Gal4/+. PP4c RNAi= 10XSTAT92E-dGFP,tub-Gal80*^*ts*^*/UAS-PP4c*^*RNAi*^*; repo-Gal4/+*. NS, nonsignificant

Although Draper is basally expressed in glia in the healthy adult brain, axon injury triggers transcriptional upregulation of *draper*, which ensures that adequate levels of the receptor are available to drive dramatic morphogenic changes in glial cell morphology and phagocytic function in the days after axotomy. Upregulation of *draper* after nerve injury requires the transcription factor STAT92E, and requisite STAT92E binding elements have been defined in the *draper* promoter.^20^ Activation of STAT92E in glia can be easily tracked in adult brains by monitoring the activation of a *10XSTAT92E-dGFP* reporter,^20^ which contains 10 tandem canonical STAT92E binding sites that control expression of a destabilized form of cytosolic GFP.^35^ To determine if STAT92E signaling requires the PP4 complex, we severed the antennal nerves of flies expressing glial *PP4c*^*RNAi*^ as well as the *10XSTAT92E-dGFP* transgene and quantified GFP levels one day postinjury. Notably, activation of *10XSTAT92E-dGFP* was indistinguishable from control animals (Figures 5a–d and f), although the robust increase in Draper protein typically observed in controls (Figures 5a, b, and e, *P*<0.0001) was inhibited in glial PP4c-depleted animals (Figures 5c, d, and e). This finding that STAT92E-dependent transcription appears unchanged in *PP4c*^*RNAi*^ animals, combined with our PP4cHA rescue experiment (Figure 4), further supports a model in which the PP4 complex is acting downstream of Draper to drive glial membrane infiltration of neuropil regions to access severed axons.

### PP4 promotes glial membrane recruitment and clearance of degenerating axons via the SOS GEF complex and Rac1

**Figure 6:**
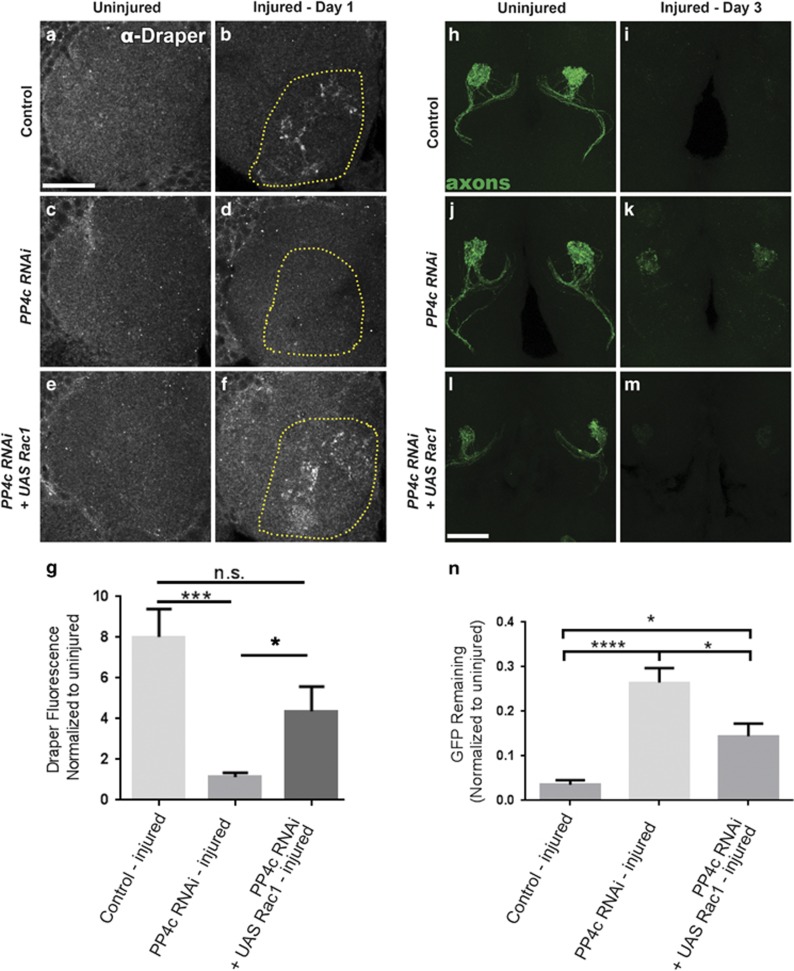
Forced glial expression of Rac1 rescues axonal clearance and Draper recruitment defects in PP4 knockdown flies. (**a–f**) Representative single z-slice (1 *μ*m) show anti-Draper fluorescence in one antennal lobe of uninjured (**a**, **c**, and **e**) and injured (**b**, **d**, and **f**) adult flies. Yellow dotted outlines show representative areas of Draper fluorescence quantified in OR85e glomeruli. (**g**) Draper fluorescence quantification in z-stack of 15 *μ*m, normalized to uninjured conditions. Uninjured Draper set at a value of 1; mean±S.E.M. plotted; one-way analysis of variance (ANOVA), ***P<0.001. (**h–m**) Representative maximum intensity projection confocal images (z-stack, 15 *μ*m) show green fluorescent protein (GFP)-labeled OR85e axonal projections (green) in antennal lobes of uninjured (**h**, **j**, and **l**) and injured (**I**, **k**, and **m**) adult flies. (**n**) GFP fluorescence quantification, normalized to uninjured condition. Uninjured GFP fluorescence set to 1; mean±S.E.M. plotted; *P<0.05 and ****P<0.0001. Scale bars=20 *μ*m. Genotypes: Control=*OR85e-mCD8::GFP,tub-Gal80*^*ts*^*/+ repo-Gal4/+. PP4c RNAi=OR85e-mCD8::GFP,tub-Gal80*^*ts*^*/UAS-PP4c*^*RNAi*^*; repo-Gal4/UAS-LacZ. PP4c RNAi+UAS-Rac1=OR85e-mCD8::GFP,tub-Gal80*^*ts*^*/UAS-PP4c*^*RNAi*^*; repo-Gal4/UAS-Rac1*. NS, nonsignificant

The Rho GTPase Rac1 is necessary for glial cell cytoskeletal remodeling and membrane recruitment to injured axons in adult *Drosophila*,^13, 16^ and *in vitro* work has shown that PP4 can modulate Rac1 activity to influence cell migration of HEK293 cells.^31^ Therefore, we wondered if PP4 might promote glial membrane recruitment to severed axons in adult *Drosophila* glia through regulation of Rac1 activity. We overexpressed wild-type Rac1 (*UAS-Rac1*) in adult glia while also knocking down PP4c by RNAi, and, interestingly, found that the Draper recruitment phenotype typically observed in *PP4c*^*RNAi*^ animals was rescued (Figures 6a–g, *P*<0.05, yellow outlines). Next, we quantified clearance of GFP+ OR85e axons after maxillary nerve injury, and found that overexpression of Rac1 in *PP4c*^*RNAi*^ flies also significantly rescued delayed removal of axonal debris (Figures 6h–n, *P*<0.05). Overexpression of *Rac1* alone did not result in faster clearance of severed axons or increased Draper accumulation after axon injury (Supplementary Figure 4). Taken together, these results suggest that the PP4 complex functions upstream of Rac1, or potentially in a partially redundant parallel pathway, to drive glial infiltration of injury sites and proper glial clearance of degenerating nerves.

**Figure 7:**
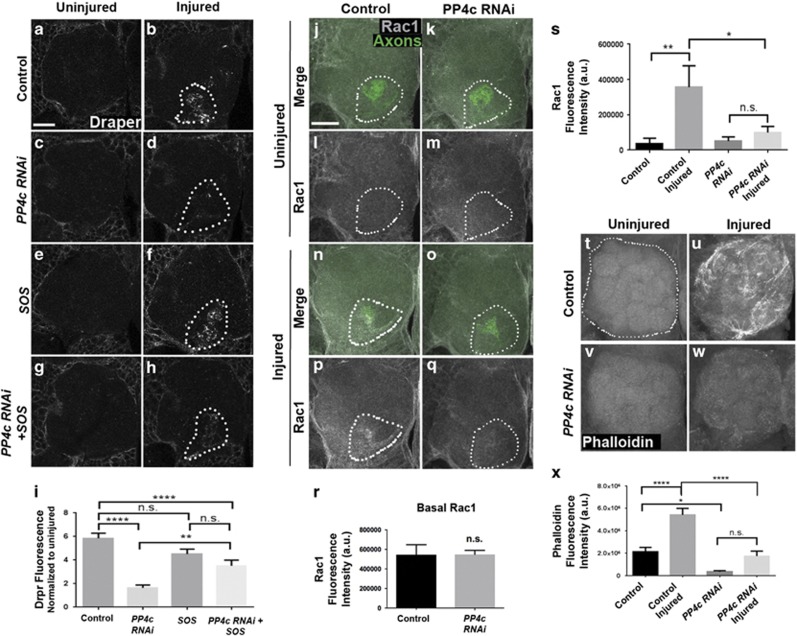
PP4 is an upstream effector of SOS and Rac1-mediated cytoskeletal remodeling. (**a–h**) Representative confocal images of Draper staining in single antennal lobes of control (**a** and **b**), PP4c RNAi (**c** and **d**), UAS-SOS (**e** and **f**), and PP4c RNAi+UAS-SOS (**g** and **h**) uninjured and injured brains. Single 1* μ*m slice shown. Dotted lines show region of interest (OR85e glomerulus) for Draper quantification. (**i**) Quantification of Draper fluorescence from experiment shown in (**a**–**h**), on 15 *μ*m Z-stack, normalized to uninjured conditions. Mean±S.E.M. plotted; one-way analysis of variance (ANOVA), Sidak's comparison test. **P<0.01, ****P<0.0001. Scale bar=20 *μ*m. Genotypes: Control=*OR85e-mCD8::GFP,tub-Gal80ts/+ repo-Gal4/+. PP4c RNAi=OR85e-mCD8::GFP,tub-Gal80ts/UAS-PP4c^RNAi^; repo-Gal4/UAS-LacZ. SOS=OR85e-mCD8::GFP,tub-Gal80ts/UAS-SOS-Myc; repo-Gal4/+. PP4c RNAi+SOS=UAS-SOS-Myc/UAS-PP4c^RNAi^; repo-Gal4/tub-Gal80ts*. (**j–q**) Representative confocal images of anti-Rac1 in single antennal lobes of control (**j**, **l**, **n**, and **p**) and PP4c RNAi (**k**, **m**, **o**, and **q**) uninjured (**j–m**) and injured (**n–q**) adult flies. Merge panels (**j**, **k**, **n**, and **o**) show anti-Rac1 (gray scale) and OR85e glomeruli (green). (**l**, **m**, **p**, and **q**) show the Rac1 channel alone. (**r**) Quantification of basal anti-Rac1 levels from cortical areas. (**s**) Quantification of anti-Rac1 fluorescence in region of interest (dotted line around 85e glomeruli) Z-stack of 15 *μ*m. Mean±S.E.M. plotted; one-way ANOVA. *P<0.05, Scale bars=20 *μ*m. Genotypes: Control=*OR85e-mCD8::GFP,tub-Gal80*^*ts*^*/+ repo-Gal4/+. PP4c RNAi=OR85e-mCD8::GFP,tub-Gal80*^*ts*^*/UAS-PP4c^RNAi^; repo-Gal4/+*. (**t–w**) Phalloidin F-actin (gray scale) staining on control and PP4c RNAi brains, uninjured (**t** and **v**) and 1 day after antennal nerve injury (**u** and **w**). Representative antennal lobes shown; 20 *μ*m z-stacks. (**x**) Phalloidin fluorescent intensity quantification. Dotted outline in (**t**) shows representative area of quantification (single antennal lobe). Mean±S.E.M. plotted; one-way ANOVA. *P<0.05 and ****P<0.0001. Scale bar: 20 *μ*m. Genotypes: Control=*OR85e-mCD8::GFP,tub-Gal80*^*ts*^*/+ repo-Gal4/+. OR85e-mCD8::GFP,tub-Gal80*^*ts*^*/UAS-PP4c^RNAi^; repo-Gal4/+*. GFP, green fluorescent protein; NS, nonsignificant

Two independent GEF complexes (DRK/DOS/SOS and Crk/Mbc/Ced-12) reportedly activate Rac1 in ensheathing glia postaxotomy.^13, 16^ To determine if PP4 is coupled to activation of the SOS GEF complex, we used *repo-Gal4* to overexpress SOS (*UAS-SOS-Myc*) and *PP4c*^*RNAi*^ flies in adult glia, severed maxillary palp nerves, and then assayed Draper accumulation on maxillary palp glomeruli that house severed axons. Interestingly, SOS overexpression significantly reversed Draper recruitment phenotypes in *PP4c*^*RNAi*^ animals (Figures 7a–i, *P*<0.01). We performed comparable experiments in an attempt to manipulate the PP4 complex and Crk/Mbc/Ced-12 in glia by coexpressing *Ced-12*^*RNAi*^ and *PP4c::HA* or coexpressing *PP4c*^*RNAi*^ and *mbc*, but these experiments resulted in lethality. Therefore, although the mechanistic connection between PP4, the Mbc GEF complex, and Rac1 in reactive glia is still unclear, our findings do highlight DRK/SOS/DOS as one key GEF complex that promotes PP4-mediated dynamics in reactive glia required for proper Draper accumulation at injury sites.

Rac1 localization is often coupled to its activity within a cell. We performed Rac1 immunostaining on PP4c glial knockdown and control brains before and one day after maxillary nerve axotomy. Significant Rac1 accumulation was visible on injured axons in control animals (Figures 7j, l, n, p, and s, *P*<0.01), but not in *PP4c*^*RNAi*^ brains (Figures 7k, m, o, q, and s). We also confirmed that PP4c depletion did not alter basal Rac1 levels in the central brain by quantifying Rac1 fluorescence in regions immediately adjacent to the antennal lobes (Figure 7r). To further explore the connection between PP4c and glial cytoskeletal remodeling after antennal nerve injury, we performed phalloidin stains to visualize filamentous actin (F-actin). One day after antennal nerve axotomy, phalloidin levels were markedly increased in the antennal lobe neuropil regions of controls (Figures 7t, u, and x, *P*<0.0001), but almost undetectable in *PP4c*^*RNAi*^ flies (Figures 7u). Collectively, these experiments indicate that PP4 does not influence basal expression of Rac1 in adult glia but instead bolster the notion that PP4 activates Rac1-mediated cytoskeletal remodeling via DOS/SOS/DRK to promote glial responses to nerve injury.

### Axotomy results in reduced nuclear Falafel expression in responding glia

**Figure 8:**
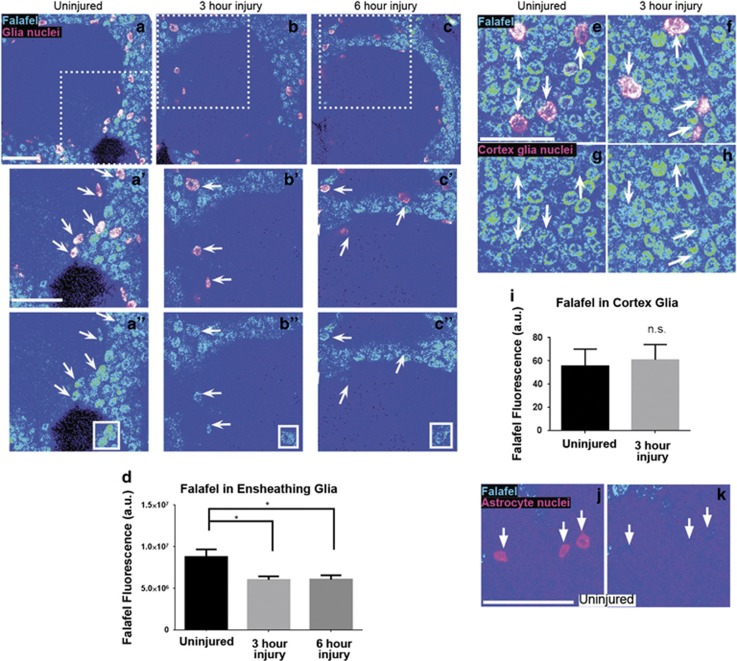
Nuclear levels of Falafel decrease in ensheathing glia after axon injury. (**a–c**) Representative confocal images of antennal lobe regions. Brains were stained with anti-Falafel and with anti-*β*-gal to visualize ensheathing glial nuclei. Dotted squares in (**a**–**c**) outline higher magnification images in (**a′**, **a″**, **b′**, **b″**, **c′**, and **c″**). White boxed regions in (**a″**–**c″**) show Falafel fluorescence in isolated glial cells. Arrows identify representative glial cells that were quantified. (**d**) Quantification of Falafel fluorescence in ensheathing glial nuclei. (**e–h**) Representative images of Falafel fluorescence in cortex glia, identified by anti-*β*-gal expression. (**i**) Quantification of Falafel fluorescence in cortex glial nuclei. (**j–k**) Representative images of Falafel fluorescence in astrocyte nuclei. Eleven micrometer of z-stacks. Mean±S.E.M. plotted; one-way analysis of variance (ANOVA). *P<0.05. Scale bar: 20 *μ*m. Genotypes: (**a–d**) *UAS-LacZ::NLS; TIFR-Gal4.* (**e–i**) *UAS-LacZ::NLS; NP2222-Gal4*. (**j** and **k**) *UAS-LacZ::NLS; alrm-Gal4*. NS, nonsignificant

The regulatory subunits Falafel and PP4r2 regulate PP4 phosphatase complex activity by influencing subcellular localization and substrate recognition.^21, 23, 29, 32^ Translocation of Falafel between the nucleus and cytoplasm to access targets for dephosphorylation has been reported in various species and cell types.^23, 24, 25, 33^ Thus, we wondered if Falafel might exit the nucleus in glia responding to axotomy to facilitate PP4 complex activity. We expressed nuclear *β*-galactosidase (*β*-gal) (*UAS-LacZ::NLS*) under the control of the ensheathing glial driver *TIFR-Gal4* to label ensheathing glial nuclei, performed antennal nerve axotomy, and then immunostained brains with anti-*β*-gal and anti-Falafel. Comparing uninjured and injured animals, we quantified nuclear Falafel levels by computationally segmenting to *β*-gal, and found that Falafel fluorescence was significantly decreased at 3 and 6 h postinjury (Figures 8a–d, *P*<0.05). Notably, we repeated this experiment labeling the nuclei of cortex glia (*NP2222-Gal4, UAS-LacZ::NLS*) adjacent to the antennal lobes, but did not detect a significant change in nuclear Falafel levels after axon injury (Figures 8e–i), indicating that Falafel location and/or levels are specifically influenced in the ensheathing glia responding to axotomy. Antennal lobe astrocytes did not express any detectable Falafel (Figure 8j and k).

## Discussion

The Draper receptor is essential for proper initiation of dynamic glial responses to axotomy in the adult *Drosophila* olfactory system.^5^ Ensheathing glia fail to infiltrate neuropil regions after olfactory nerve axotomy in *draper* mutant animals due to inadequate Rac1 activity.^16^ The mechanisms that couple activation of Draper to Rac1-mediated cytoskeletal remodeling, glial recruitment to injury sites, and phagocytic clearance of severed axons are poorly understood. Our results now implicate the PP4 phosphatase complex as a critical molecular effector that functions downstream of Draper to activate the DOS/SOS/DRK GEF complex and Rac1 to promote dynamic cytoskeletal rearrangements in glia responding to axotomy.

The PP4 phosphatase complex is implicated in diverse cellular functions, including mitosis, DNA strand break repair, and differentiation.^21, 22, 23, 24, 25, 26, 27, 28, 29, 30, 31, 32, 32, 36, 37, 38, 39^ Our work now highlights a previously unexplored role for PP4 in governing innate glial immune responses to neurodegeneration and poses interesting questions for future efforts aimed at understanding precisely how PP4 activity promotes cell migration. We show that forced SOS GEF expression rescues loss of PP4c (Figures 7a–i), implicating the SOS GEF complex as one key effector downstream of PP4 required for proper Rac1 activity in responding glia. To the best of our knowledge, direct biochemical interactions between PP4 and GEF complexes have not been reported. Thus, it is unlikely that the SOS/DOS/DRK complex is directly targeted by PP4 in glia; future screening efforts will be required to delineate the complete signaling pathway that couples PP4 to the SOS complex. We also cannot rule out the possibility that additional GEF complexes (e.g. Crk/Mbc/Ced-12) converge on glial Rac1 to coordinate the assorted dynamic reactions required for glia to access and dispose of degenerating axonal debris. Finally, because glial activation (e.g. recruitment of glial membranes) and phagocytic clearance are tightly coupled, it is unclear if delayed removal of axonal material in PP4-depleted flies is exclusively a result of inadequate glial membrane invasion of the neuropil or if the PP4 complex independently regulates phagocytic internalization of axonal debris via SOS/Rac1- or a Rac1-independent mechanism.

In *Drosophila*, Falafel and PP4r2 are the exclusive regulatory subunits that associate with the catalytic subunit PP4c to form a functional trimeric complex. Mammalian genomes contain six or more genes that encode regulatory PP4 subunits, which enhances the capacity for combinatorial control over PP4 activity across cell types and biological states.^21^ PP4 complex activity can be regulated, in part, by subcellular localization of the regulatory subunits. For example, in starving *Dictyostelium*, the Falafel homolog SMEK translocates from the cytoplasm into the nucleus where it activates PP4c to facilitate cell stress responses.^25^ *Drosophila* PP4 complex components also cycle between the nucleus and cytoplasm of proliferating neural precursors, which is essential for PP4 to associate selectively with key targets in the nucleus and cytoplasm and facilitate the proper distribution of cell fate determinants during asymmetric cell division.^23, 32^ Our observation that nuclear levels of Falafel decrease significantly in ensheathing glial cells surrounding the antennal lobes within hours after olfactory nerve injury suggests that expression and/or function of PP4 is modified in the glia as innate glial immune responses are elicited. We favor the model that Falafel is translocated out of the nucleus, but we cannot exclude the possibility that it becomes incorporated into a complex that hinders antibody accessibility or becomes degraded. We did not detect a significant increase in Falafel levels in glial cytoplasm postinjury, but this may reflect *in vivo* imaging limitations while attempting to visualize low concentrations of Falafel distributed throughout the cell.

The serine/threonine PP4 complex was recently identified as a requisite factor for proper immune responses in T cells, B cells, and macrophages,^40, 41, 42^ including proliferation and immune gene induction. Although the specific role of PP4 in glial cell immunity has not been investigated, increased PP4 expression has been reported in glial tumors, suggesting a connection between PP4 function and glial cell invasiveness.^43^ Draper is a highly conserved glial receptor essential for glial clearance of damaged and dying neurons across species. The mammalian homolog, MEGF10, is required for glial clearance of apoptotic neurons, as well as developmental axonal/synaptic pruning.^4, 5, 10, 11, 12, 13, 14, 15, 16, 20, 44, 45, 46, 47, 48, 49, 50^ The high conservation of GEF/Rac1-mediated control of cell migration is also well documented.^51, 52, 53, 54^ Our work now reveals that the PP4 phosphatase complex unifies these two conserved molecular signaling pathways in the context of glial immunity and may also provide new molecular insight into glial tumor cell migration.

## Materials and methods

### *Drosophila* stocks

The following *Drosophila* strains were used: w^1118^ (BL5905). repo-Gal4/TM3.^12^ OR85e-mCD8::GFP/CyO.^55^ w; Sp/CyO; repo-LexA, LexAop-mCD2::RFP/TM3.^56^ UAS-LacZ::NLS,tub-Gal80^ts^/CyO (BL 7108). w^1118^; UAS-mCD8::GFP; UAS-LacZ::NLS. w^1118^; UAS-lacZ::NLS (BL3955). TIFR-Gal4.^9^ alrm-Gal4.^11^ y^1^ v^1^; P(TRiP. JF02802)qattP2/TM3, Sb (falafel^RNAi^, BL31961). w; UAS-Rac1 (BL6293). P(GD9561)v25317 (PP4c^RNAi^, VDRC). P(KK100895)VIE-260B (PP4r2^RNAi^, VDRC). UAS-SOS::myc/Cyo; TM2/Sb (a kind gift from Yang and Bashaw^57^). NP2222.^56^ UAS-mbc.^58^ UAS-PP4-19c::3XHA (FlyORF, F001063).^59^ y^1^ v^1^; P(TRiP. HMJ22104)attP40 (Ced-12^RNAi^, BL58153).

### Adult fly brain injury, dissection, and immunostaining

Maxillary palp and antennal ablations were performed on adult flies as described previously.^5^ Maxillary or antennal nerves were severed by removing maxillary palps or third antennal segments, respectively, with forceps. One to five days postinjury, heads were removed and fixed in 4% PFA+0.01% Triton-X for 15 min, followed by washing with 1 × PBS+0.01% Triton-X for 3 × 2 min. Brains were dissected in 1 × PBS+0.01% Triton-X in glass well plates, and then fixed in 4% PFA+0.1% Triton-X for 15 min. Brains were washed in 1 × PBS+0.1% Triton-X for 3 × 2 min, and then placed in primary antibody diluted in 1 × PBS+0.1% Triton-X overnight at 4 °C. Brains were then washed in 1 × PBS+0.1% Triton-X for 3 × 2 min, and then placed in secondary antibody diluted in 1 × PBS+0.1% Triton-X for 2 h at room temperature. Brains were washed in 1 × PBS+0.1% Triton-X for 3 × 2 min, and then placed in CitiFluor CFM-I mounting media (Electron Microscopy Sciences, Hatfield, PA, USA) for 30 min before being mounted on glass slides. Flies with *tub*-*Gal80*^*ts*^ were raised at 22 °C, shifted to the restrictive temperature 30 °C posteclosion for 3–7 days, injured, and then returned to 30 °C until dissection. Each genotype had equal amounts of male and female flies. The following antibodies were used: mouse anti-Draper (1:400, Developmental Studies Hybridoma Bank, Iowa City, IA, USA); rat anti-Falafel (1:1000, a kind gift from Bill Chia); chicken anti-GFP (1:1000, Life Technologies, Carlsbad, CA, USA); mouse anti-Repo (1:10, Developmental Studies Hybridoma Bank); phalloidin-TRITC (1:250, Sigma, St. Louis, MO, USA; no. P1951); mouse anti-Rac1 (1:250, BD Biosciences, San Jose, CA, USA; no. 61650); chicken anti-β-gal (1:2000; Abcam, Cambridge, MA, USA; no. ab9361). All secondary antibodies (Jackson Immunoresearch, West Grove, PA, USA) were used at a concentration of 1:400.

### Western blot analysis

Central brains (optic lobes manually removed) were homogenized in 4 ml 1xLB (loading buffer) per brain. Lysates were loaded into 4–20% Tris-glycine gels (Lonza, Allendale, NJ, USA) and transferred to Immobilon-FL (Millipore, Billerica, MA, USA). Blots were probed with rabbit anti-Draper (1 : 1000, kind gift from Marc Freeman) and sheep anti-tubulin (Cytoskeleton, Denver, CO, USA; no. ATN02). Blots were incubated with primary antibodies overnight at 4 °C, washed 3 × with 1xPBS+0.01%Tween-20, and then incubated with secondary antibodies (713-625-147 and 711-655-152, from Jackson Immunoresearch, West Grove, PA, USA) for 2 h at room temperature. Blots were then washed 3 × with 1xPBS+0.01% Tween-20 and 1 × with 1xPBS. Blots were imaged on LI-COR Odyssey CLx Quantitative Western Blot Imaging System, and data were quantified with Li-COR Image Studio software (Li-COR, Lincoln, NE, USA).

### Confocal microscopy and image analysis

Brains were mounted in CFM-I mounting medium and imaged using a Zeiss LSM 710 confocal microscope (Zeiss, Thornwood, NY, USA). Brains were imaged in 1 *μ*m steps with a x40 1.4 NA oil immersion plan apochromatic lens. Brains in a single experiment were imaged in the same day on the same slide with the same confocal settings.

### Axonal clearance

Quantification of GFP was performed in 3D volumes of OR85e glomeruli (15 *μ*m z-stacks).

### Draper recruitment

Draper pixel intensity was quantified in 3D regions of interest in the antennal lobe of 15 *μ*m z-stacks (see dotted outline in Figure 2). These dotted regions were selected because they correspond to OR85e− glomeruli, which was visualized by the introduction of a OR85e-mCD8::GFP transgene.

### Membrane expansion

Glial membrane expansion after antennal ablation was measured as RFP+ intensity in 3D regions of interest in the antennal lobe of 15 *μ*m z-stacks.

### Falafel translocation

Falafel translocation experiments were quantified by segmenting to the β-gal-positive nuclei (either ensheathing glia or cortex glia) and then measuring mean Falafel fluorescence in these glial nuclei only using Volocity. All image analysis was performed using Volocity image analysis software (Perkin-Elmer, Hopkinton, MA, USA).

### Statistics

All statistics were performed in GraphPad Prism 6 (GraphPad, La Jolla, CA, USA). *T*-tests and one-way ANOVAs were performed as appropriate (see figure legends). All experiments were repeated in full at least three times and *post hoc* power tests were run to ensure sample size adequacy. Experiments were not blinded. *N* for each genotype for each experiment: Figures 1a–d) control: 17, control injured: 12, Falafel: 23, Falafel injured: 19, Figures 1f–k) control: 10, control injured: 12, PP4c: 20, PP4c injured: 20, PP4r2: 14, PP4r2 injured: 16. Figures 1m and n) control: 6, Falafel: 6. Figure 2: control: 18, control injured: 18, Falafel: 12, Falafel injured: 16, PP4c: 12, PP4c injured: 13, PP4r2: 20, PP4r2 injured: 12. Figure 3: Antennal Injury: control: 4, control injured: 6, PP4c: 18, PP4c injured: 24. Maxillary Palp Injury: N: control: 13, control injured: 16, PP4c: 4, PP4c injured: 6. Figure 4: control: 20, control injured: 18, Draper RNAi: 16, Draper RNAi injured: 20, Draper RNAi+PP4cHA: 4, Draper RNAi+PP4cHA injured: 3, PP4cHA: 6, PP4cHA injured: 5. Figure 5: control: 19, control injured: 22, PP4c: 14, PP4c injured: 8. Figure 6: control: 19, control injured: 19, PP4c: 14, PP4c injured: 12, PP4c+Rac1: 10, PP4c+Rac1 injured: 10. Figures 7a–h) control: 26, control injured: 27, PP4c: 16, PP4c injured: 20, SOS: 8, SOS injured: 10, PP4c+SOS: 15, PP4c+SOS injured: 12. Figure 7j and q) control: 22, injured: 25, PP4c: 11, PP4c injured: 28. Figures 7t–w) control: 18, injured: 24, PP4c: 21, PP4c injured: 26. Figures 8a–c) uninjured: 13, 3 h injured: 12, 6 h injured: 12. Figures 8e–h) uninjured: 20, 3 h injury: 17.
