# Supplementary material for: Protein phosphatase 4 coordinates glial membrane recruitment and phagocytic clearance of degenerating axons in Drosophila
Source: Cell Death Dis. 2017 Feb 23;8(2):e2623–. doi: 10.1038/cddis.2017.40 (PMC5386485; doi:10.1038/cddis.2017.40)
Supplement: Supplementary Information [file cddis201740x1.pdf]

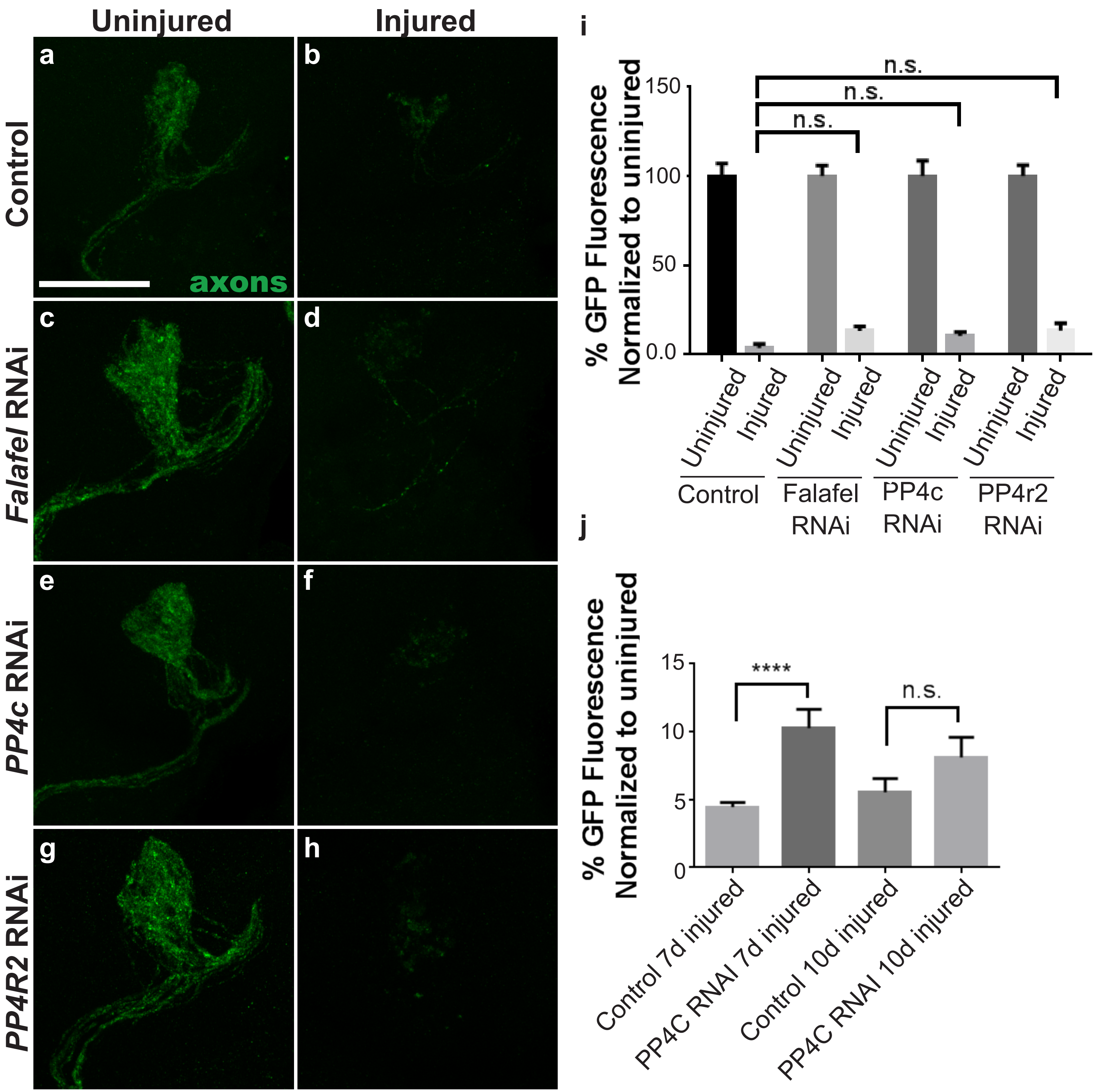

Supplemental Figure 1: Axonal clearance is unaltered in room temperature controls for adult-specific RNAi lines. a-h: Representative maximum intensity projection confocal images (z-stack, 15 um) show GFP-labeled OR85e axonal projections (green) in antennal lobes of uninjured (a,c,e,g) and injured (b,d,f,h) adult flies. i: Quantification of OR85e axonal debris GFP fluorescence normalized to uninjured conditions. Uninjured GFP fluorescence values set at 1. j: Clearance timeline. Quantification from room temperature day 7 and day 10 injuries of OR85e axonal debris GFP fluorescence normalized to uninjured. Z-stack: 15um. n>9 for each experiment; mean ± s.e.m. plotted; 1-way ANOVA. Scale bars = 20 um. Genotypes: OR85e-mCD8::GFP,tub-Gal80ts/+; RepoGal4/+. OR85e-mCD8::GFP,tub-Gal80ts/+; RepoGal4/UAS-Falafel RNAi. OR85e-mCD8::GFP,tub-Gal80ts/UAS-PP4c RNAi; RepoGal4/+. OR85e-mCD8::GFP,tub-Gal80ts/UAS-PP4r2 RNAi; RepoGal4/+

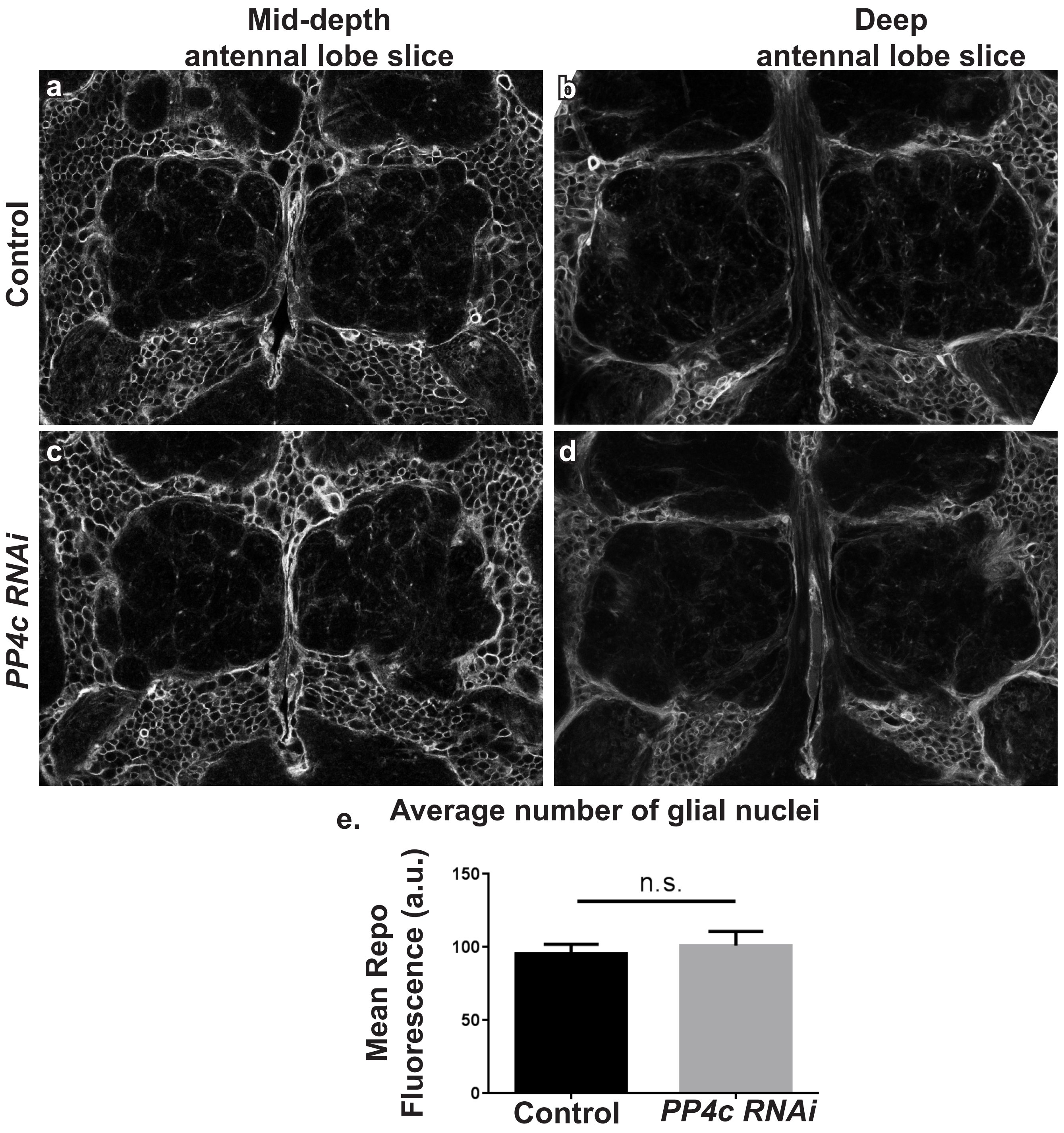

Supplemental Figure 2: Basal glial membranes are morphologically similar in PP4c knockdowns. Representative antennal lobe single slices (1um) show basal glial membrane-RFP in greyscale in control (a,b) and PP4c RNAi (c,d) adult flies. Images from mid-depth (a,c) antennal lobes shown and from deep (posterior) (b,d) antennal lobes. Scale bars = 20 um. Genotypes: OR85e-mCD8::GFP,tub-Gal80ts /+; RepoGal4/ Repo-LexA, LexAop-mCD2::RFP. OR85e-mCD8::GFP,tub-Gal80ts/PP4c RNAi; RepoGal4/ Repo-LexA, LexAop-mCD2::RFP.
e: Average number of glial cells does not change in PP4c knockdown compared to controls. Mean Repo (glial nuclei) fluorescence quantified. N: control: 12, PP4c RNAi: 11. Genotypes: OR85e-mCD8::GFP,tub-Gal80ts/+; Repo-Gal4/+. OR85e-mCD8::GFP,tub-Gal80ts/PP4c RNAi; Repo-Gal4/+

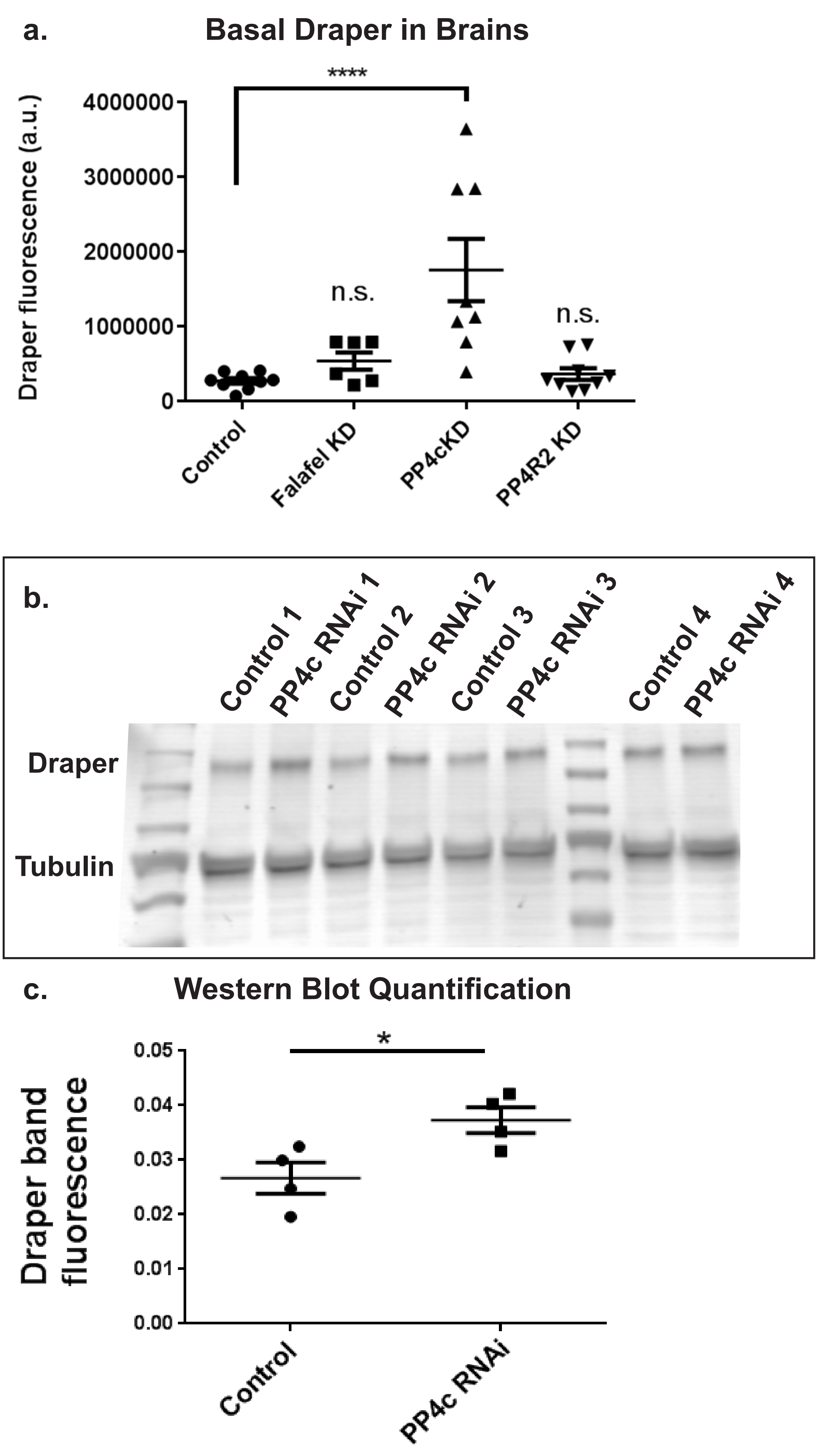

Supplemental Figure 3: Analysis of basal Draper levels in PP4 subunit knockdown flies. a: Quantification of basal Draper fluorescence from cortical areas in control brains compared to glial expression of Falafel RNAi, PP4c RNAi, or PP4r2 RNAi. b: Western blot of uninjured control and PP4c RNAi central brain tissue probed for Draper and tubulin. c: Quantification of Draper bands on Western blot, normalized to tubulin bands, N: 14 heads/ sample. Genotypes: Control = OR85e-mCD8::GFP,tub-Gal80ts/+; repo-Gal4/+. Falafel KD = OR85e-mCD8::GFP,tub-Gal80ts/+; repo-Gal4/UAS-Falafel RNAi. PP4c KD= OR85e-mCD8::GFP,tub-Gal80ts/UAS-PP4c RNAi; repo-Gal4/+. PP4r2 KD = OR85e-mCD8::GFP,tub-Gal80ts/ UAS-PP4r2 RNAi; repo-Gal4/+.

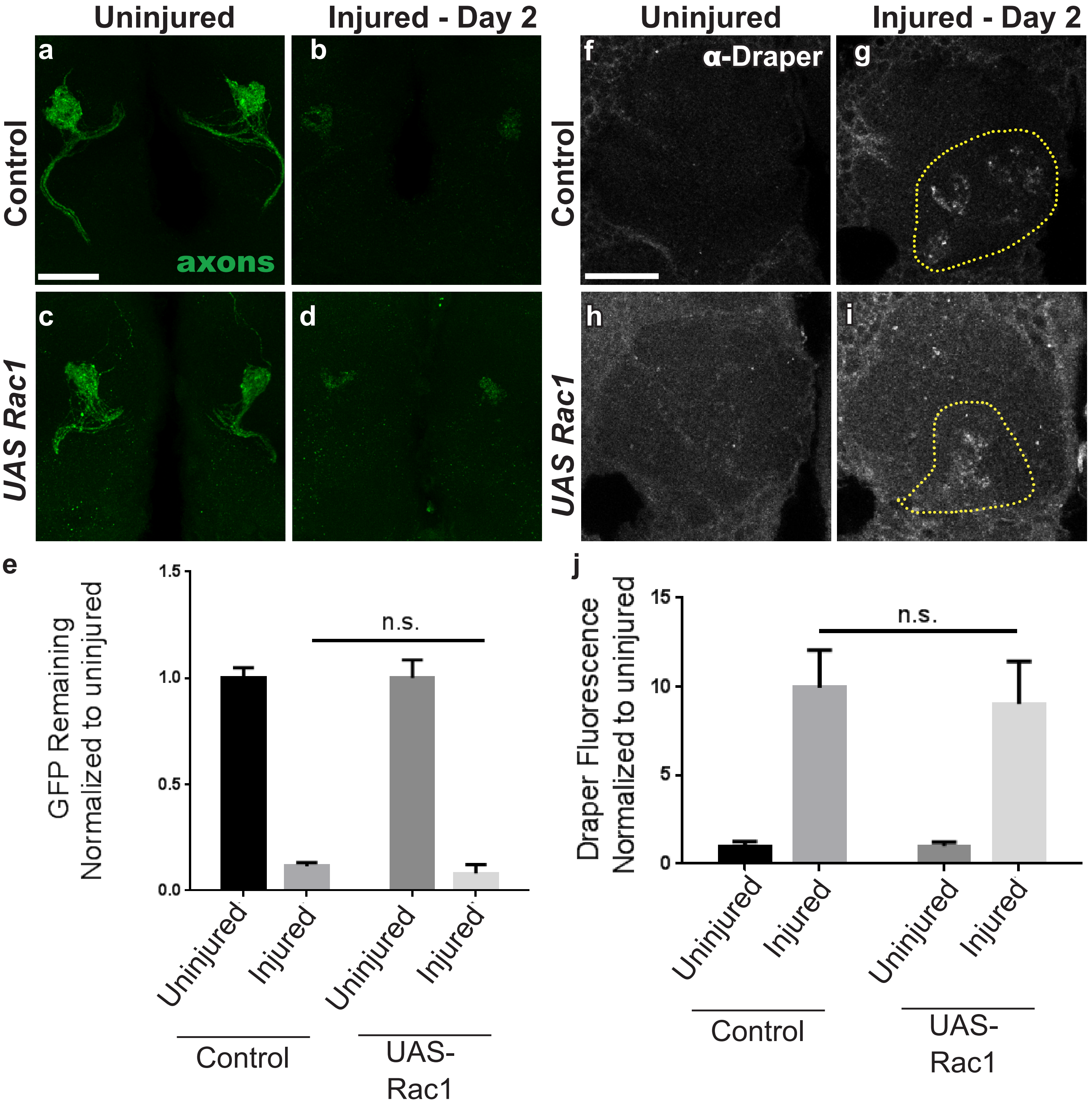

Supplemental Figure 4: Rac1 overexpression does not alter axonal clearance or Draper recruitment. a-d: Representative maximum intensity projection confocal images (z-stack, 15 um) show GFP-labeled OR85e axonal projections (green) in antennal lobes of uninjured (a,c) and injured (b,d) adult flies. e: Quantification of OR85e axonal debris GFP fluorescence normalized to uninjured conditions in 15um z-stacks. Uninjured GFP fluorescence values set at 1; n>11 for each experiment; mean ± s.e.m. plotted. Scale bars = 20 um. f-i: Representative single z-slice (1um) show anti-Draper fluorescence (grey) in one antennal lobe of uninjured (f,h) and injured (g,i) adult flies. Yellow dotted outlines show representative areas of Draper fluorescence quantified in OR85e glomeruli. j: Draper fluorescence quantified, normalized to uninjured conditions in 15um z-stacks. Uninjured Draper set at a value of 1; n>9 for each experiment; mean ± s.e.m. plotted; 1-way ANOVA. Scale bars = 20 um. Genotypes: OR85e-mCD8::GFP,tub-Gal80ts/+; RepoGal4/+. OR85e-mCD8::GFP,tub-Gal80ts+; RepoGal4/UAS-Rac1
